# Validation of a composite outcome measure for inpatient psychiatry using scales from the interRAI-MH

**DOI:** 10.3389/fpsyt.2025.1486734

**Published:** 2025-02-06

**Authors:** Howard E. Barbaree, Christopher Perlman, Elke Ham, Gregory P. Brown, John P. Hirdes

**Affiliations:** ^1^ Department of Psychiatry, University of Toronto, Toronto, ON, Canada; ^2^ School of Public Health Sciences, University of Waterloo, Waterloo, ON, Canada; ^3^ Waypoint Research Institute, Waypoint Centre for Mental Health Care, Penetanguishene, ON, Canada; ^4^ Criminal Justice Department, Nipissing University, North Bay, ON, Canada

**Keywords:** inpatient psychiatry, outcome measures, health care quality, interRAI mental health, content validity, concurrent validity, predictive validity, construct validity

## Abstract

**Background:**

Inpatient psychiatry is a critical service in a community-based care system for persons with serious mental illness (SMI). Currently, there are few generally accepted or widely used outcomes to assess the effectiveness of inpatient treatment.

**Method:**

Following a Donabedian Model of Health Care Quality, we utilized eight scales from the RAI Mental Health assessment to derive a clinician-scored outcome measure consisting of 4 domains (Psychosis, Depression, Impairment, and Aggression). We combined subscales measuring these domains into a Composite Measure. We used this measure to assess the entire population (N=719) of our large specialized mental health hospital at the beginning (T1) and end (T2) of three months in the hospital (or admission to discharge in shorter stays). We evaluated the content validity of the measure by comparing items and scales with a list of putative contributors to hospital admission (symptoms and complications). To evaluate concurrent validity, we compared mean scores among hospital units with varying lengths of stay and clinical complexity (acute versus chronic versus complex chronic). We used ROC analysis to evaluate the CIIMHS’s ability to predict discharge from the hospital. To evaluate construct validity, we examined the measure’s responsiveness to changes among patients after treatment in the hospital.

**Results:**

We found strong evidence for all four kinds of validity.

**Conclusions:**

The composite measure represents a valid measure of inpatient mental health status and will serve as a valuable measure of the quality of care for inpatient psychiatry.

## Introduction

A Serious Mental Illness (SMI) interferes with a person’s life and ability to function (1). SMIs include but are not limited to Major Depression, Schizophrenia, Schizo-affective Disorder, and Bipolar Disorder. Persons with SMI face severe threats to their health, well-being, and autonomy. Beyond their symptoms, including depressed mood, hallucinations, delusions, and mania, they may face additional complications in varying degrees of severity across various SMIs that add substantially to the disease burden. Specifically, there are five notable complications. Persons with SMI can become isolated (2), both because their symptoms and behavior repel others but also due to their waning capacity for meaningful social interaction (3). Social isolation reduces the likelihood of support in the community and aggravates mental illness (3–5), creating a harmful feedback loop. They become impaired, losing their ability to function in society, including deficits in self-care (6). Persons with SMI are at higher risk for suicide and other forms of self-harm (7, 8). Persons with SMI may engage in aggression/violence (9, 10). As much as 50% of persons with SMI have a co-morbid Substance Use Disorder (SUD) (11), and SUDs are both a mental disorder and a complication of SMI.

Depending on life circumstances, the eventual outcomes of SMI can be devastating for some individuals. Isolation and impairment can lead to loss of employment and income (12), leading to poverty, poor diet (13), and homelessness (14, 15). Their physical health can be affected (16, 17), and poor physical health aggravates mental illness (18), creating a second vicious circle. Aggression/violence often leads to criminal charges and detention in the correctional (19) or forensic mental health systems (20, 21). The result can be physical, psychological, and social deterioration, including shortened life expectancy (22, 23).

The disease process is punctuated by crises when symptoms are most severe, and the risk for harm is greatest. Often, mental health crises are precipitated by intoxication by alcohol or some other psychoactive substance (24). Deterioration, risk of injury, and harm to others impose an obligation on society to prevent these outcomes. Crises often lead to hospitalization to minimize or prevent harm or further deterioration. Historical developments such as deinstitutionalization (25), the widespread acceptance of the recovery model (26), and the development of more comprehensive community services have left inpatient psychiatry with a diminished but critical role in the continuum of psychiatric care: amelioration of symptoms and complications and rapid return to the community.

In this environment, the quality of mental health care provided in hospitals, both in effectiveness and efficiency, becomes of critical importance and widespread interest. Current discussions of healthcare quality have evolved from Donabedian’s conceptual framework (27–29), which considers quality of care proportional to the improvement in health status brought about by medical intervention. Donabedian’s framework defined “health status” as a metric that quantifies the patient’s overall level of health at a particular point in time. When the practitioner assesses the patient’s health status before and after the provision of care, the magnitude of the positive difference is an outcome reflecting the quality of care.

Donabedian’s model conceived of quality of care as involving three elements. Structure included the physical infrastructure, materials, and human resources required to provide care. Process was the work of medical professionals providing care. Hospital quality improvement programs have easily identified structures and processes, incorporating them into their balanced scorecards. However, hospitals can improve the third element, implementing meaningful Outcomes. Given the importance of outcomes to establishing inpatient mental health care quality, it is surprising how few measures have been made available. The Health of the Nation Outcome Scales (HoNOS) (30, 31) is a clinician-rated measure for use with adults in contact with mental health services. It contains 22 items assessing behavior, impairment, symptoms, and social functioning. Each item is scored on a 5-point scale from zero (no problem) to 5 (severe problem). It is used in the UK, Australia, New Zealand, and many parts of Europe. Its use in North America has been limited.

The present work explores the development of a new outcome measure based on the Resident Assessment Instrument-Mental Health (RAI-MH) (32). interRAI (33) is a not-for-profit international network that develops and implements assessment methods to support improved quality of care among vulnerable persons with complex needs in health and social service settings. The Resident Assessment Instrument-Mental Health (RAI-MH) (32), comprised of 460 items, was developed for inpatient mental health services, including acute, forensic, long-stay, and geriatric psychiatry. It was created through a systematic multi-step process including literature reviews, consultations with clinicians and experts, inclusion of items from other interRAI instruments, expert working group sessions, surveys of front-line staff, debriefing sessions after reliability testing, focus groups, and nursing retreats. As a result, the RAI-MH has 19 content areas assessing mental and physical health, status, functioning, cognitive performance, substance use, support systems, and relationships, as well as health service use. In 2005, the Government of Ontario mandated the RAI-MH for use in all government-funded inpatient psychiatric services in Ontario. Since 2005, according to protocol, the RAI-MH has been administered to psychiatric inpatients in Ontario on admission, discharge, and every three months for long-stay patients or whenever there was a significant change in clinical status. As of 2020, over 1.4 million assessments have been completed on over 320,000 unique individuals in Canada (34). Though designed for use in Inpatient Psychiatry, interRAI has adapted the RAI-MH for use in other mental health care settings, including Community Mental Health (interRAI CMH) (35), Emergency Psychiatry (interRAI-ESP) (36), and Child and Youth Mental Health (interRAI ChYMH) (37). interRAI refers to these instruments as “integrated” because they share a common language, conceptual basis, clinical emphasis, collection method, core elements, and care planning protocols. The RAI-MH has been updated and improved, and the new instrument is called the interRAI-MH (38). For the current study, we used the RAI-MH, but the revised and improved instrument contains all the elements that we used for our research. In this paper, we use the terms RAI-MH and interRAI-MH interchangeably.

To examine the feasibility of developing mental health quality indicators (MHQIs) based on the RAI-MH, Perlman and colleagues (39) performed retrospective analyses on two large data sets from over 70 facilities in Ontario. They derived potential MHQIs by examining the empirical improvement patterns in the severity of depressive symptoms (DSI) and cognitive performance (CPS) levels across facilities. While mean scores on these scales showed substantial variation among facilities, these authors concluded that deriving MHQIs using data from the RAI-MH is a feasible approach to assessing the quality of care. Tolonen and colleagues (40) used a multidimensional model of mental illness and various scales from the interRAI-CMH to evaluate outcomes for participants in a residential psychiatric rehabilitation program. They scored the scales before and after a 29-month (median) duration of treatment. Depression, mania, and positive and negative symptoms of psychosis showed significant pre-post improvement. The performance of daily activities and risks for self-harm and harm to others improved from before to after rehabilitation.

The Donabedian model represents a person’s multidimensional health status as a two-dimensional matrix. The vertical dimension quantifies the level of function or performance (health status). The horizontal dimension represents various functional areas (symptoms, impairment, isolation, risk to self, and danger to the public) that contribute to health status. Donabedian suggested that an average status across the different functional areas could represent an individual’s overall health status. We will refer to these functional areas as “components” of mental health status and their combination as the Composite Index of Inpatient Mental Health Status (CIIMHS).

## Methods

### Participants

The data analyses reported here used a sample (N=719) that includes all patients in hospital care at a large tertiary care facility during one fiscal year. Their average age was 43 years. Nearly three-quarters were male (73%). Most were never married (69%); 14% were married or had a live-in partner at the time of their admission to the hospital, and 17% were widowed, separated, or divorced. Almost all were English-speaking (99%). Even though more than half (54%) had graduated from high school, and 22% had some post-secondary education, only 8% were employed, and 13% had no income. All others were on a pension, social assistance, or disability.

The research described in this manuscript was subject to Waypoint’s Research Ethics Board Approval of an application entitled “The Development of Clinical Outcome Measures based on the interRAI-MH among clinical programs at Waypoint Centre for Mental Health Care Certificate # CRRA#12.03.01.

### Setting

Waypoint Centre for Mental Health Care is a large psychiatric hospital and forensic mental health research facility in Penetanguishene, Ontario, Canada. The hospital is one of four specialty mental health care facilities in Ontario. It provides extensive acute and longer-term psychiatric inpatient and outpatient services to the surrounding region and has Ontario’s only high-secure forensic mental health program.

**Table 1** presents salient information regarding Waypoint’s ten inpatient programs, including patients’ legal status (whether patients are voluntary, civilly committed, or forensic), length of stay, clinical complexity, bed count, level of security, whether the program admits female patients and the number of patients who received care in the program during the fiscal year.

**Table 1:** Hospital programs and mean program CIIMHS scores.

Legal Status	Length of Stay^1^	Clinical Complexity	Discharge to Community?	# of Beds	Level of Security^2^	Co-Ed?	N	Mean CIIMHS	Post^3^ Hoc	SD CIIMHS
Forensic	Chronic	PD^4^	No	40	Double Locked	No	42	*2.24*	*a*	2.42
Civil	Acute	SMI^6^+SUD^7^	No	20	Open	Yes	59	*2.78*	*a*	2.42
Civil^5^	Acute	SMI^6^	Yes	20	Open	Yes	241	*3.44*	*a*	3.12
Civil	Chronic	SPMI^8^ Simple	Yes	40	Locked	Yes	60	*4.78*	*a,b*	3.03
Forensic	Chronic	SPMI^8^Simple	Yes	20	Locked	Yes	28	*5.38*	*a.b*	3.61
Forensic	Acute	SMI^6^	No	40	Double Locked	No	140	*7.29*	*b*	4.63
Forensic	Chronic	SPMI^8^ Simple	No	40	Double Locked	No	41	*7.59*	*b*	3.85
Civil	Chronic	SPMI^8^ Complex^9^	Yes	16	Locked	Yes	24	*8.31*	*b,c*	3.94
Forensic	Chronic	SPMI^8^ Complex^9^	No	40	Double Locked	No	43	*11.3*	*c,d*	4.46
Civil	Chronic	SPMI^8^ Complex^10^	No	20	Locked	Yes	41	*12.4*	*d*	4.07
**Total: Whole Hospital**				296			719	5.64		4.63

Programs are rank ordered in the table according to the mean CIIMS score at T1.

^1^Short term <30 days and Medium term 30-150 days = Acute; Long Term >150 days = Chronic.

^2^Double Locked includes a Sally Port.

^3^Post-hoc test: Program means with common letters are not significantly different.

^4^Personality Disorder.

^5^Mixed = both voluntary and involuntary (Mental Health Act Ontario).

^6^Serious Mental Illness.

^7^Substance Use Disorder.

^8^Serious and Persistent Mental Illness.

^9^Intellectual/Developmental Disabilities.

^10^Dementia.

A Forensic patient is an individual charged with a criminal offense with a confirmed or suspected mental disorder who is sent to Waypoint for assessment or treatment by a court or a review board under the terms in the Criminal Code of Canada (CCC). Civil patients either volunteer for inpatient care or are committed to the hospital on an involuntary basis under the terms of the Mental Health Act (Ontario).

The hospital divides its programs into categories depending on the length of stay and the complexity of the mental illnesses typically seen in the program. Acute programs care for patients over a short period (generally less than thirty days} or for slightly more extended periods (31-150 days) because of fixed-program curricula or a fixed period of hospitalization specified in an order from the court. Chronic programs care for patients over an extended period (>150 days). Further, the hospital divides its Chronic programs according to the complexity of the diagnoses of their typical patients. Simple Chronic programs serve patients with severe and persistent mental illness (SPMI). Complex Chronic programs serve patients with SPMI, plus complicating additional diagnoses (e.g., Dementia or Intellectual/Developmental Disabilities).

The Forensic Personality Disorders Program serves men brought into the forensic mental health system before 1992 when Canadian courts often found a person who committed a criminal offense Not Guilty by Reason of Insanity (NGRI) because of a personality disorder alone (PD; usually Antisocial). Due to their high risk to public safety, authorities have refused to transfer these patients to lower levels of security or release them to the community. As a result, these PD-only patients have remained at Waypoint. Since 1992, according to the Criminal Code of Canada (CCC), the court must hear evidence that the accused person has a diagnosis of SMI (including psychosis) to be admitted to the forensic system. Consequently, the number of PD-only patients has remained static. The hospital’s Personality Disorder Program serves men who have been in the hospital for a long time but do not suffer from an SMI, providing our study with a convenient no-SMI comparison group.

### Materials

Hirdes and colleagues (41) listed 17 RAI-MH scales that yield a score based on an RAI-MH assessment. From among these, we chose scales that could be categorized into one of seven putative causes of inpatient admission (symptoms of depression, symptoms of psychosis, isolation, impairment, risk of self-harm, aggression/violence, and substance abuse). Seeking scales that would be sensitive to the changes brought about by treatment, we eliminated scales that contained a preponderance of static or historical items that would not change with treatment. Structural validity (42) is related to content validity and relates to the statistical interdependencies among scale scores on a multidimensional instrument and whether or not these subscales reflect coherent and distinct underlying factors. Accordingly, we were interested in deriving CIIMHS components that were relatively independent of one another (orthogonal). To that end, we eliminated scales whose computation depended on scores from other scales. **Table 2** presents the results of our scale selection. We chose eight scales, as indicated in the table. The psychometric properties of these scales are good (43, 44).

**Table 2:** Components of the CIIMHS.

Mental Disorders	Symptoms	Complications	RAI MH Scales		Components of CIIMHS
Serious Mental Illness:	of Psychosis		Positive Symptoms^1^	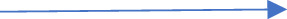	Psychosis
Major Depression				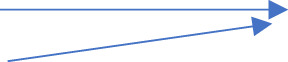	
Schizophrenia	of Depression		Depression Severity^2^		Depression
Schizo-Affective Disorder		Social Isolation	Social Withdrawal^3^		
Bipolar Disorder					
		Cognitive Impairment	Cognitive Performance^4^	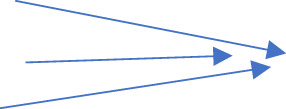	
		Functional Impairment,	Activities of Daily Living^5^		Impairment
		including Deficits in	Instrumental Activities^6^		
		Self Care	of Daily Living		
		Risk of Harm to Self	Severity of Self Harm Scale is static	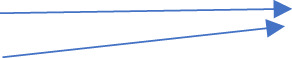	Risk of Self Injury
		Risk of Harm to Others	Aggressive Behavior ^7^		Aggression
			Violence Sum ^8^		
Substance Use Disorder	of Intoxication	Substance Abuse	CAGE scale is static		Substance Abuse
	of Withdrawal				

Abbreviated items for the 8 scales.

^1^PSS, Hallucinations, delusions, abnormal thought, inflated self-worth, hyperarousal, pressured speech, abnormal movements.

^2^DSI, Sad, pained, worried facial expression; made negative statements, self-depreciation, guilt or shame, hopelessness.

^3^SWS, Decreased energy, blunted affect, lack of pleasure, withdrawal from activities, lack of motivation, reduced interaction.

^4^CPS, Short-term memory, cognitive skills for daily decision-making, making oneself understood.

^5^ADL, Personal hygiene, walking, wheeling, toilet use, eating.

^6^IADL, Meal preparation, managing medications, transportation, managing finances, phone use.

^7^ABS, Verbal abuse, physical abuse, socially inappropriate/disruptive behavior, resistance to care.

^8^VS, Violent to others, intimidation of others or threatened violence, violent ideation.

### Procedure

Waypoint Decision Support supplied a data file derived from RAI-MH assessments for all 1,491 assessments completed at the hospital during the relevant fiscal year. For each assessment, the file contained a unique but anonymous identifying number for each patient, their program assignment at the time of the assessment, the dates for each assessment, the assessment type (admission, discharge, quarterly, or change in status), and item scores for the eight scales described above.

There were 719 unique patients provided inpatient care at Waypoint during the relevant fiscal year. First, we sought every patient’s most recent RAI-MH assessment before the end of each of the four fiscal quarters. We will refer to these as assessments at T2. Then, for each patient, we sought the immediate previous assessment. We will refer to these as assessments at T1. For patients with numerous T1-T2 pairings, we chose a single T1-T2 pair on the following bases. First, we eliminated a pairing if the patient was transferred to a different program between T1 and T2, unless the patient had only one T1T2 pairing. Second, we chose the pair with the most extended interval between T1 and T2. If there were two or more pairings with roughly equal intervals, we chose the earlier pairing. Finally, to ensure that the choice of T1 T2 pairings was independent of scale scores, we finalized the eventual data set of T1 T2 pairings before the computation of scale scores.

### Calculation of the CIIMHS and component scores

**Table 2** presents the conceptual framework we used to determine CIIMHS components. Column 1 presents the mental disorders. Column 2 presents the symptoms of depression and psychosis. Column 3 presents the complications of these mental disorders (impairment, isolation, risk of harm to self, and aggression/violence). Column 3 lists the eight dynamic scales derived from the RAI-MH sorted into these categories.

Hirdes and colleagues (41) provide directions for calculating scale scores. All statistical analyses described here were done with SPSS Version 29. To evaluate internal consistency (reliability), Coefficient Alpha was calculated for each of the eight scales. **Table 3** presents Coefficient Alpha for each scale, separately for T1 and T2, as well as for the eventual component scores. Additionally, the table includes Alphas for scales previously published by Hirdes et al. (2020) (34).

**Table 3:** Cronbach's Alpha (N-719) at T1 and T2 for seven RAI-MH Scales and the CIIMHS component scales.

	Alpha @ T1			Alpha @ T2			Hirdes et al 2020 (34)
	Alpha	Lower 95%CI	Upper 95%CI	Alpha	Lower 95%CI	Upper 95%CI	
RAI-MH Scales^1^							
Aggressive Behavior Scale (ABS)	0.76	0.73	0.79	0.77	0.74	0.79	0.77
Cognitive Performance Scale CPS)	0.61	0.58	0.65	0.67	0.64	0.70	xx
Depression Severity Index (DSI)	0.59	0.55	0.64	0.51	0.46	0.56	0.76
Instrumental Activiies of Daily Living (IADL)	0.94	0.94	0.95	0.96	0.95	0.96	0.94
Positive Symptoms Scale (PSS)	0.65	0.61	0.68	0.65	0.62	0.69	0.71
Social Withdrawal Scale (SWS)	0.77	0.73	0.79	0.76	0.73	0.78	xx
Violence Sum (VS)	0.84	0.82	0.86	0.89	0.88	0.91	xx
CIIMHS Components^2^							
Psychosis	0.65	0.61	0.68	0.65	0.62	0.69	
Depression	0.73	0.70	0.76	0.71	0.67	0.73	
Impairment	0.91	0.90	0.91	0.91	0.90	0.92	
Aggression	0.81	0.80	0.83	0.85	0.83	0.86	

^1^ADL is scored as a hierarchical item where internal consistency is not relevant so it is not included here.

^2^Alphas based on all items contributing to the Component.

We conducted several Principal Components Analyses (PCA; Factor Analyses) of the eight RAI MH scales. Extraction was based on Eigenvalues greater than 1.00 and Varimax Rotation with Kaiser Normalization. All rotations converged in 5 or fewer iterations. The top panel of **Table 4** presents the results of our PCAs. The first three columns show the results from T1, identifying three factors accounting for 68% of the variance. The first factor reflected functional impairment in daily tasks (ADL, IADL) and cognitive (CPS) performance. The second included the scales involving aggression and violence (ABS, VS) together with psychotic symptoms (PSS). The third included scales reflecting depressed mood (DSI, SWS). The second three columns in the top panel present the PCA results from T2. This PCA found three factors, accounting for 71% of the variance. Again, the first factor reflected functional impairment in daily tasks (ADL, IADL) and cognition (CPS). But here, the second factor included scales reflecting depressed mood (DSI, SWS) and psychotic symptoms (PSS). At T2, the third factor included aggression and violence scales (ABS, VS). Therefore, the eight scales’ factor structure was unstable from T1 to T2. Specifically, the scale PSS was associated with violence and aggression at T1 and with depressed mood at T2. The instability of the factor structure from T1 to T2 presents the risk that factors might indicate changes from T1 to T2 when, in actuality, the factors represent a different set of mental health components at T1 versus T2. We recalculated each of these two PCAs, excluding the PSS scale. These results are presented in the lower panel on the right-hand side of **Table 4**. As can be seen, the factor structure is identical at T1 and T2, with the factors accounting for 74% and 76% of the variance, respectively. At T1 and T2, the first factor reflected functional impairment in performing daily tasks (ADL, IADL) and cognition (CPS). The second factor included the aggression and violence scales (ABS, VS). The third included scales reflecting depressed mood (DSI, SWS).

**Table 4:** Principal Components Analysis of RAI MH scales separately at T1 and T2.

Panel 1 (Includes PSS)	Rotated				Rotated		
	Factor Loadings T1				Factor Loadings T2		
Factor	1	2	3		1	2	3
8 RAI MH Scales							
ADL	0.898			ADL	0.914		
IADL	0.878			CPS	0.875		
CPS	0.842			IADL	0.865		
VS		0.798		**PSS**		**0.776**	
**PSS**		**0.688**		DSI		0.773	
ABS		0.674		SWS		0.554	
DSI			0.787	VS			0.900
SWS			0.755	ABS			0.805

Panel 2 (Ecludes PSS)	Rotated				Rotated		
	Factor Loadings T1				Factor Loadings T2		
Factor	1	2	3		1	2	3
7 RAI MH Scales							
ADL	0.886			ADL	0.902		
IADL	0.878			IADL	0.881		
CPS	0.853			CPS	0.880		
VS		0.907		VS		0.907	
ABS		0.760		ABS		0.813	
SWS			0.797	SWS			0.793
DSI			0.793	DSI			0.729

Panel 1 (above) presents the PCA results for all 8 RAI MH scales at T1 (left) and T2 (right).

The PSS item is presented in bold to draw attention to the fact that it is unstable from T1 to T2.

Panel 2 (below) presents the PCA results for the 7 items with the item PSS excluded.

In this case, the factor structure is stable from T1 to T2.

The distributions of these eight scale scores differed widely in statistical properties (means, standard deviations, and ranges). If we had combined raw scale scores to calculate the composite score, scales with greater ranges would have added greater weight to the composite score. To avoid this, we standardized scale scores to the Z distribution so that each scale had a mean of zero and a standard deviation of 1.00. We made transformations of the standardized scores to make the numbers more “clinician-friendly.” We eliminated the negative numbers by transforming z scores so that each scale ranged from 0.00 to 10.00.

As a result of our data analysis above (PCAs), we considered the following as mental health components. The first would include the scales ADL, IADL, and CPS; the component would be named “impairment.” The second would consist of the ABS and VS scales and be called “aggression.” The third would include DSI and SWS scales, called “depression.” The PSS scale would represent our measure of “Psychosis.”. We calculated component scales as follows: Impairment = (ADL_0-10_+IADL_0-10_+ CPS_0-10_)/3; Aggression = (ABS_0-10_ +VS0_0-10_)/2; Depression = (DSI_0-10_ + VS_0-10_)/2; and Psychosis = PSS_0-10_. We calculated the CIIMHS as the sum of the 4 component scores and scaled the component measures to have similar ranges to the composite by multiplying each component score by 5. **Table 5** presents descriptive statistics for the CIIMHS and its components separately for T1 and T2.

**Table 5:** Descriptive statistics for the CIIMHS and component scores at T1 and T2 (N=719).

	Maximum	Mean	Std. Deviation	Skewness	Kurtosis
At T1					
** CIIMHS**	25.19	5.64	4.63	1.02	0.82
** Psychosis**	50.04	6.62	8.71	1.71	3.48
** Depression**	45.05	5.92	7.13	1.82	4.01
** Impairment**	50.09	7.64	11.35	1.98	3.22
** Aggression**	50.03	7.97	9.68	1.40	1.73
At T2					
** CIIMHS**	24.67	4.34	4.63	1.26	1.19
** Psychosis**	49.97	5.12	8.46	2.15	5.03
** Depression**	34.70	4.05	5.96	2.00	4.64
** Impairment**	49.98	6.32	10.50	2.13	3.85
** Aggression**	49.98	6.23	8.78	1.95	4.29

Minimums are zero except for rounding error.

### Validity, hypotheses, and statistical analysis

Content Validity evaluates the extent to which a test measures a representative sample of the subject matter under investigation and how well a test covers all relevant aspects of the construct it aims to measure (45). Concurrent validity refers to how closely a new measure relates to a known criterion or gold standard. We hypothesized that our new measure would significantly relate to hospital program assignment (length of stay, complexity of diagnosis). Predictive Validity refers to a measure’s ability to predict the occurrence of a particular outcome. We hypothesized that the CIIMHS would predict hospital discharge. Construct validity concerns how well a measure represents or reflects a concept that is not directly measurable (46). The ultimate purpose of developing outcome measures is to support treatment program evaluation and improvement. Construct validity relates to the outcome measure’s responsiveness to change in health status when it occurs. We hypothesized that our new measure would reflect pre (T1) to post (T2) changes when they occur.

#### Concurrent validity

Hypothesis 1. Mean program CIIMHS scores will follow the order of PD-only patients (lowest mean scores), acute patients, simple chronic patients, and complex chronic patients (highest mean scores). Between groups ANOVA will be used to test this hypothesis, with planned orthogonal comparisons followed by the Tukey *Post Hoc* test. Planned orthogonal comparisons included the PD-only group versus all other programs combined, acute programs versus chronic programs, and simple chronic programs versus complex chronic programs.

Hypothesis 2. Discharged patients will have lower mean CIIMHS than current patients, and the CIIMHS will predict discharge from the hospital.

#### Predictive validity

Hypothesis 3. We evaluate our ability to predict future behavior using a statistical analysis called Receiver Operator Characteristics (ROC) (47, 48). The ROC curve plots the trade-off between true and false positives across a test’s measurement range. An AUC value of.50 indicates a ‘chance’ relationship between predictor and outcome, while an AUC value of 1.00 represents a ‘perfect’ prediction (rare). Good AUC values typically range between.65 and.80, with AUCs above.80 considered large. The AUC represents the probability that the CIIMHS score for a randomly chosen discharged patient is lower than that of a randomly chosen current patient.

#### Construct validity

Hypothesis 4. The mean CIIMHS will reduce from T1 to T2 throughout the hospital.

Hypothesis 5. Acute programs will show significant reductions in mean CIIMHS over the study interval, but chronic programs will not.

Hypothesis 6. Discharged patients will show a larger reduction in mean CIIMHS than current patients.

Mixed Between-Groups Within-Ss (T1 v T2) ANOVAs will be used to test these hypotheses. The single factor within-subjects ANOVA provides three estimates of variance (Sums of Squares) (1): the difference between the means at T1 and T2 (Effect) (2); differences among patients’ marginal scores (T1 + T2); and (3) error.


$${\text{SS}}_{\text{Total}}={\text{ SS}}_{\text{Effect}}+{\text{ SS}}_{\text{Patients}}+{\text{ SS}}_{\text{Error}}$$

The variance estimate for error represents the consistency of change from T1 to T2 across patients. The more similar the change is from T1 to T2 across patients, the smaller the error.

We will make important comparisons between programs’ T1 vs. T2 effect sizes. The Effect Size (ES) (49) is calculated as:


$$\text{Partial Eta Squared }\left({\text{η}}_{\text{p}}^{2}\right)\;={\text{SS}}_{\text{effect}}/\left({\text{SS}}_{\text{effect}}+{\text{ SS}}_{\text{error}}\right)$$

Therefore, two factors determine the effect size. The larger the difference between the means at T1 and T2 and the more consistent the T1 to T2 changes among patients, the larger the ES. This makes the Partial Eta Squared an ideal statistical model for quality of care as defined by the Donabedian model.

## Results

### Content validity of the CIIMHS

**Table 2** presents the conceptual framework used to evaluate the content validity of the CIIMHS. We were unable to find scales representing the Risk of Self-harm and Substance Abuse that met our selection criteria. The Severity of Self-Harm scale included the PSS, DSI, and CPS in its calculation, and the History of Suicide Attempt (a static item) is a major component in its calculation. The CAGE scale contained a preponderance of static items. Five component scales derived from 8 scales from the RAI-MH quantify the severity or extent of the five remaining putative causes of mental health crises. Accordingly, our analysis of Content Validity finds that the CIIMHS contains 5 of 7 relevant components. As seen in **Table 5**, concerning the weighting of the five components of the composite measure, with most scales ranging from 0-50, the mean component scores mainly were within 2.00 of the mean CIIMHS, indicating roughly equal weighting.

### Concurrent validity

**Table 1** orders the hospital programs according to their mean CIIMHS scores.

Hypothesis 1. Hospital Programs differed widely in mean CIIMHS scores, F_(9,709)_ = 52.25, p<.001. The Forensic PD program (no-SMI) had the lowest mean CIIMHS at T1 (2.24), and the Civil Chronic SPMI + Dementia program had the highest mean CIIMHS (12.40). Concerning our planned comparisons, the PD-only program had a significantly lower CIIMHS mean score at T1 than other programs combined, F_(1,709)_ = 67.09, p<.001. Among the remaining programs, Chronic programs had significantly higher CIIMHS mean scores at T1 than Acute programs, F_(1,709)_ = 141.89, p<.001, and Complex Chronic programs had higher CIIMHS mean scores at T1 than Simple Chronic programs, F_(1,709)_ = 93.72, p<.001 The 10th column in **Table 1** presents the results of the Tukey *Post Hoc* Test (Alpha = 0.01). These results were all in line with our predictions.

Hypothesis 2. Using data (N=353) from 4 hospital programs that routinely discharge to the community (for other programs, discharges involved transfers to other hospital programs, either at Waypoint or other hospitals in the province), the mean CIIMHS score (3.53) of discharged patients (N=257) was significantly lower than the mean CIIMHS score (5.82) of current inpatients (N=96), F_(1,351)_ =33.62, p<.001.

### Predictive validity

**Figure 1:**
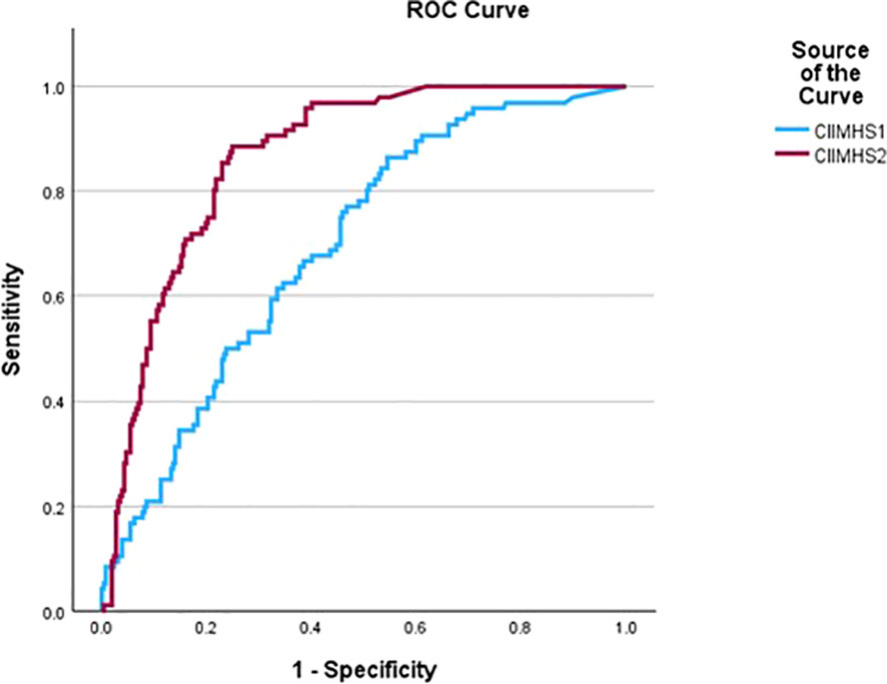
Predictive validity. ROC Curves for the prediction of patient discharge by CIIMHS (N=353). The lower curve is for CIMHS at T1 (AUC=.69; 95% CI = .64-.75) and the upper curve is for CIMHS at T2 (AUC=.87; 95% CI = .83-.90).

Hypothesis 3. The results of the ROC analysis are presented graphically in **Figure 1**. CIIMHS at T1 yields an AUC of 0.69 (95% CI = .64-.75). This AUC was statistically significant (p<.001), and its numerical value indicated a good level of prediction accuracy. CIIMHS at T2 yielded an AUC of.87 (95% CI.83-.90), was statistically significant (p<.001), and is regarded as a high level of prediction accuracy. Since each AUC value is outside the other’s CIs, the CIIMHS at T2 is a significantly better predictor than the CIIMHS at T1.

### Construct validity

On average, the time between T1 and T2 was 55.33 days and ranged between 1 and 180 days. (Mode = 92 days; Median = 68 days). The time between assessments varied among programs, F_(13, 1477)_ = 175.12, p<.001. The Civil Acute SMI program had the shortest mean interval between assessments (13.5 days). The Forensic Acute SMI program and the Voluntary Acute SMI + SUD program had the second shortest mean interval between assessments (58.7 and 69.5 days, respectively). The remaining programs had mean intervals between assessments from 79.8 to 92.9 days. Usually, a researcher would be concerned that shorter intervals between assessments would compromise a program’s apparent effectiveness since shorter intervals provide less time for treatment effects to be realized. However, as will be seen, the programs with shorter intervals between assessments showed the most significant improvement in mental health status.

**Table 6** presents mean CIIMHS scores for all hospital programs at T1 and T2. We used hospital programs as the grouping factor in a 2 (T1 vs T2) within by 10 between groups design.

**Table 6:** Statistical comparisons T1 versus T2 by hospital program.

Forensic/ Civil	Length of Stay	Clinical Complexity	N	Mean	SD	Mean	SD	F	df	sig	Partial eta^2^
				CIMHS	CIMHS	CIMHS	CIMHS				
				at T1^1^	at T1	at T2	at T2				
Forensic	Chronic	PD	42	2.24	2.42	1.75	2.16	5.12	1,41	<.05	0.112
Civil	Acute	SMI+SUD	59	2.78	2.42	1.14	2.05	20.55	1,58	<.001	0.262
Civil	Acute	SMI	241	3.44	3.12	1.54	2.55	73.54	1,240	<.001	0.235
Civil	Chronic	SPMI Simple	60	4.78	3.04	4.18	2.92	1.81	1,59	ns	0.030
Forensic	Chronic	SPMI Simple	28	5.38	3.61	5.11	2.71	<1	1,27	ns	0.008
Forensic	Acute	SMI	140	7.29	4.64	5.52	4.12	21.81	1,139	<.001	0.136
Forensic	Chronic	SPMI Simple	41	7.59	3.86	7.23	4.46	<1	1,40	ns	0.019
Civil	Chronic	SPMI Complex^2^	24	8.31	3.94	7.96	3.53	<1	1,23	ns	0.021
Forensic	Chronic	SPMI Complex^2^	43	11.30	4.46	11.31	3.70	<1	1,42	ns	0.000
Civil	Chronic	SPMI Complex^3^	41	12.40	4.07	11.48	4.64	1.99	1,40	ns	0.047
**Total: Whole Hospital**			719	5.64	4.63	4.34	4.63	95.02	1,718	<.001	0.117

^1^Programs are ordered according to Mean CIIMHS at T1.

^2^Intellectual/Developmental Disability.

^3^Dementia.

Hypothesis 4. Over the whole hospital, as predicted, there was a reduction in CIIMHS from T1 (mean = 5.64) to T2 (mean = 4.34), F_(1,718)_ =95.02, p<.001; Partial Eta Squared (η_p_^2^) = 0.117, a medium effect size (50, 51).

Hypothesis 5. The changes from T1 to T2 were not uniform across hospital programs, as the interaction between T1 vs T2 and programs was significant, F_(1,709)_ = 3.15, p<.001. As predicted, Acute programs significantly reduced CIIMHS scores from T1 to T2, but Chronic programs did not.

**Table 6** presents separate within-subjects ANOVAs for the individual programs. The Civil Acute SMI+SUD program shows a decline in mean CIIMHS score from T1 (2.78) to T2 (1.14) that was statistically significant, F_(1,58)_ = 20.55, p<.001, with a large effect size (η_p_^2^) = 0.262. The Civil Acute SMI program showed a significant decline in mean CIIMHS scores from T1 (3.44) to T2 (1.54), F _(1,240)_ = 73.54, p<.001, with a large effect size (η_p_^2^) of .235. The Forensic Acute Assessment program showed a decline from T1 (7.29) to T2 (5.52), F_(1,139)_ = 21.81, p<.001, with a medium effect size of (η_p_^2^) = 0.136.

The only significant reduction in the CIIMHS among the Chronic programs occurred in the Forensic PD program, F_(1,41)_ = 5.12, p<.05, with a medium effect size (η_p_^2^) of.112, due to a significant reduction in the Aggression component.

Hypothesis 6. To test our prediction concerning discharged versus current patients, we selected data from patients (N=353) in programs that routinely discharged patients into the community. Within subjects ANOVA, comparing mean CIIMHS scores at T1 and T2 indicated that for patients not discharged from the hospital, the mean CIIMHS score at T2 (5.50) was not different from the mean CIIMHS score at T1 (5.82). For discharged patients, the mean CIIMHS score at T2 (1.66) was significantly reduced from that at T1 (3.53). The reduction in discharged patients was significantly greater than in current patients, F_(1,351)_ = 14.97, p<.001.

## Discussion

A Donabedian model conceptualized outcomes for inpatient psychiatry, construing inpatient mental health status as a multidimensional entity with six components, each a precursor and putative contributory cause of emergency admission to a psychiatric hospital. These were depression, psychosis, impairment, aggression/violence, self-harm, and substance use. To compute an outcome measure, we chose eight scales from the interRAI-MH based on their ability to reflect an individual’s level on one of these dimensions and their ability to reflect a change in level when it occurred. Four component measures were computed after averaging correlated measures. Based on the four measures, the CIIMHS was examined for content validity and tested for concurrent, predictive, and construct validity using a large sample of inpatients in a mental health specialty hospital. We found strong evidence for validity in all four spheres.

Regarding content validity, the CIIMHS included four of the six content areas specified above, providing a reasonably representative sample. The content validity of the CIIMHS can be evaluated by comparison with other empirically evaluated outcome measures. The HoNOS (30, 31) has very similar item content to the CIIMS. Of its 12 items, 6 have an obvious equivalent item among the 8 RAI-MH items used in the present study, and 4 pertain to issues not necessarily pertinent to inpatient care for SMI. The remaining two deal with substance abuse and self-harm, which were not included in our measure, as explained above. The basis-32 (52) is a self-report outcome measure to assess mental health treatment outcomes. It contains five of our six primary symptom and functioning domains, including psychosis, depression, addictive behavior, social relations, and daily living and role functioning.

Regarding concurrent validity, we predicted differences among hospital programs in terms of the severity of mental illness and then calculated statistical comparisons. Hospital programs’ mean CIIMHS scores accorded with their predicted mental health status. Acute programs had lower mean scores than chronic programs. Simple Chronic programs had lower mean scores than Complex Chronic programs, and the Personality Disorder program had lower scores than those serving SMI patients. Patients discharged during the quarter had lower scores than patients who remained in the hospital. Regarding predictive validity, CIIMHS scores significantly predicted discharge from the hospital at T1 and T2, with more accurate prediction at T2.

Concerning construct validity, the change in the hospital’s CIIMHS mean score from T1 to T2 was statistically significant overall. Moreover, as predicted, we demonstrated that acute programs were more likely to significantly reduce CIIMHS scores than chronic programs. Patients discharged during the quarter had more significant decreases in mean CIIMHS scores from T1 to T2 than those who remained in the hospital. The only Chronic Program that showed a significant reduction in CIIMHS was the PD Program. This reduction was due to a significant reduction in the Aggression/Violence component. Hospital programs provide continuous interventions to reduce violence, which may explain this outcome.

The three Acute programs showed significant reductions in CIIMHS. These reductions support the validity of the CIIMHS as an outcome measure. The Civil/Voluntary Acute SMI program admits patients from regional ERs and provides evidence-based psychiatric treatment. Staffed by fully qualified psychiatrists and psychiatric nurses, patients receive proven psychotherapeutic medications and supportive care from allied health professionals. The Forensic Acute SMI program admits patients from the courts for assessments of fitness to stand trial or criminal responsibility or for treatment to make fit in preparation for a return to court. The primary treatment is proven antipsychotic medication.

Waypoint designed the Voluntary Acute SMI+SUD program in 2005 as an integrated mental illness and substance abuse treatment based on evidence-based programs for Concurrent Disorders (Dual Diagnosis) described in the empirical literature. The hospital developed new manuals and trained all program clinicians in several empirically supported clinical interventions, including CBT. McKee and colleagues (53) used a quasi-experimental wait-list control design to evaluate the new program. Eighty-six participants who completed the program showed clinically significant improvements in mental health symptoms, acquisition of knowledge and skills, higher self-esteem, and higher satisfaction with the program compared with the wait-list control group. Our findings are consistent with those of McKee et al.

The primary purpose of outcome studies is to evaluate program effectiveness by comparing outcomes over time and between different treatment conditions. Comparing programs in terms of their statistical effects could be done by looking at the reduction in mean CIIMHS from T1 to T2. An alternative is the Effect Size (Partial Eta Squared). In the present study, the three Acute programs’ changes from T1 to T2 are ranked similarly using differences between means or effect sizes: Civil SMI+SUD > Civil SMI > Forensic SMI. However, a similar ranking would not necessarily be obtained in all circumstances since the ES depends additionally on the consistency among patients in their changes from T1 to T2. Notably, the ES allows comparisons among groups assessed with different instruments using different scales. Partial Eta Squared (η_p_^2^) was calculated from 11,044 inferential statistics reflecting treatment effect sizes reported in the *Journal of Counseling Psychology* from 1970 to 1979 and resulted in a median value of.08 (54).

Chronic SPMI programs did not exhibit statistically significant changes from T1 to T2. The time between T1 and T2 may be an essential variable. In the study reported here, the average time between T1 and T2 for Chronic programs was between 80 and 90 days. Careful examination of **Table 4** reveals that the Civil and Forensic SPMI Simple Chronic programs show changes from T1 to T2 that are approaching levels of traditional statistical significance. Evaluating change over a longer interval may improve outcomes for these groups. Tolonon and colleagues (40) described a long-term rehabilitation program for young adults with Severe and Persistent Mental Disorders. Over a treatment period of (median length) 29 months, patients demonstrated reduced depressive symptoms, lowered risk of harm to self and others, and increased ADLs. Similarly, Van Kranenburg and associates (55) described a long-term treatment (over four years) of homeless persons suffering from Severe and Persistent Mental Illness and Substance Use Disorders in which participants demonstrated an increase in Global Psychosocial Functioning, reductions in risk of harm to self and others, reduced symptoms and an increase in skills for daily living. These authors provided effect sizes of 0.19 to 0.33 per annum and 0.42 and 0.73 over two years.

The CIIMHS represents a reasonable assessment of inpatient mental health status. However, the four CIIMHS components will not represent the breadth of relevant mental health status in all clinical venues. The CIIMHS does not include a scale representing anxiety, a frequent component of mental illness. Anxiety is a common experience for inpatients but is a rare cause of hospital admission by itself (56). The absence of a scale representing recovery or other community-relevant issues would be problematic in many settings. Indicators for recovery are dealt with in the interRAI Self-reported Quality of Life Survey for Mental Health and Addictions (57, 58). An outpatient or community setting would require different components in a composite measure.

The CIIMHS provides a standardized assessment to be used consistently in all hospital programs, allowing program managers and the hospital administration to make meaningful program comparisons. However, specialized programs may want to supplement their outcome measures with specialized instruments. For example, inpatient programs treating SUDs will want to include outcome measures relating to substance abuse, and forensic programs will want to have outcome measures relating to criminal behavior (59). Standardized measures do not imply that they reflect a complete picture of outcomes for all programs.

The findings reported here have broad and immediate applicability. As mentioned above, since 2005, according to protocol, the RAI-MH has been administered to psychiatric inpatients in Ontario on admission, discharge, and every three months for long-stay patients or whenever there was a significant change in clinical status. These findings could be replicated easily with data readily available in every psychiatric inpatient unit in Ontario. In addition, as mentioned above, the RAI-MH has been adapted to other mental health care settings, including Community Mental Health (35), Emergency Psychiatry (36), and Child and Youth Mental Health (37). These instruments contain the items our study used to derive similar outcome measures in these settings.

Finally, it’s important to say that while these results support the validity of the measures, they have not demonstrated the effectiveness of any intervention or treatment. Further studies using adequate controls to eliminate potential confounding variables (placebo effect, passage of time, change in environment) are required. The current results validate a measure that could be used in appropriately designed studies examining the effectiveness of inpatient treatment.

One problematic aspect of this approach to outcome assessment is the number of steps and time it takes to compute the CIIMHS and its components. We are developing software to make these calculations less burdensome.

From a research perspective, one drawback of the study is that data were acquired from clinicians’ scoring of the instruments as part of their regular clinical duties. Had the scoring been done as part of a rigorous research protocol, the reliability of these scales might have been better. On the other hand, our data reflect what can reasonably be done to assess the quality of care based on records available in many settings.

In summary, our validation of the CIIMHS was successful. These results have encouraged the implementation of the CIIMHS as a Routine Outcome Monitor (ROM) (60) at Waypoint. This outcome measure has been included with the structure and process QIs on the hospital’s balanced scorecard. These outcomes allow the hospital to evaluate its efforts to improve the quality of patient care over the long term.

## Data Availability

The raw data supporting the conclusions of this article will be made available by the authors, without undue reservation.

## References

[1] McGinty EE Baller J Azrin ST Juliano-Bult D Daumit GL. Quality of medical care for persons with serious mental illness: A comprehensive review. In: Schizophrenia research. New York: Elsevier, vol. 165. (2015). doi: 10.1016/j.schres.2015.04.010 PMC4670551 25936686

[2] Wang J Lloyd-Evans B Giacco D Forsyth R Nebo C Mann F. Social isolation in mental health: a conceptual and methodological review. Soc Psychiatry Psychiatr Epidemiol. (2017) 52:969–77. doi: 10.1007/s00127-017-1446-1 PMC5702385 29080941

[3] Brandt L Liu S Helm C Heinz A. The effects of social isolation stress and discrimination on mental health. Trans Psychiatry. (2022) 12:398. doi: 10.1038/s41398-022-02178-4 PMC9490697 36130935

[4] Pancani L Marinucci M Aureli N Riva P. Forced social isolation and mental health: A study on 1,006 italians under COVID-19 lockdown. Front Psychol. (2021) 12:663799. doi: 10.3389/fpsyg.2021.663799 34093358 PMC8175900

[5] Pietrabissa G Simpson SG. Psychological consequences of social isolation during COVID-19 outbreak. Front Psychol. (2020) 11:2201. doi: 10.3389/fpsyg.2020.02201 33013572 PMC7513674

[6] Chen C Chen Y Huang Q Yan S Zhu J. Self-care ability of patients with severe mental disorders: based on community patients investigation in beijing, China. Front Public Health. (2022) 10:847098. doi: 10.3389/fpubh.2022.847098 PMC9198226 35719645

[7] Brådvik L. Suicide risk and mental disorders. Int J Environ Res Public Health. (2018) 15:2028. doi: 10.3390/ijerph15092028 30227658 PMC6165520

[8] Singhal A Ross J Seminog O Hawton K Goldacre MJ. Risk of self-harm and suicide in people with specific psychiatric and physical disorders: Comparisons between disorders using English national record linkage. J R Soc Med. (2014) 107:194–204. doi: 10.1177/0141076814522033 PMC4023515 24526464

[9] Fazel. S Gulati G Linsell L Geddes. JR Grann &M. Schizophrenia and violence: Systematic review and meta-analysis. PloS Med. (2009) 6. doi: 10.1371/journal.pmed.1000120 PMC2718581 19668362

[10] Swanson JW McGinty EE Fazel S Mays VM. Mental illness and reduction of gun violence and suicide: Bringing epidemiologic research to policy. Ann Epidemiol. (2015) 25:366–76. doi: 10.1016/j.annepidem.2014.03.004 PMC4211925 24861430

[11] Ross S Peselow E. Co-occurring psychotic and addictive disorders: Neurobiology and diagnosis. Clin Neuropharmacology. (2012) 35:235–43. doi: 10.1097/WNF.0b013e318261e193 22986797

[12] Fukuura Y Shigematsu Y. The work ability of people with mental illnesses: A conceptual analysis. Int J Environ Res Public Health. (2021) 18:10172. doi: 10.3390/ijerph181910172 34639474 PMC8508570

[13] Sparling TM Cheng B Deeney M Santoso MV Pfeiffer E Emerson JA. Global mental health and nutrition: moving toward a convergent research agenda. Front Public Health. (2021) 9:722290. doi: 10.3389/fpubh.2021.722290 34722437 PMC8548935

[14] Scott J. Homelessness and mental illness. Br J Psychiatry. (1993) 162:314–24. doi: 10.1192/bjp.162.3.314 8453425

[15] Sullivan G Burnam A Koegel P. Pathways to homelessness among the mentally ill. Soc Psychiatry Psychiatr Epidemiol. (2000) 35:444–50. doi: 10.1007/s001270050262 11127718

[16] Momen NC Plana-Ripoll O Agerbo E Benros ME Børglum AD Christensen MK. Association between mental disorders and subsequent medical conditions. New Engl J Med. (2020) 382:1721–31. doi: 10.1056/nejmoa1915784 PMC7261506 32348643

[17] Vaingankar JA Chong SA Abdin E Siva Kumar FD Chua BY Sambasivam R. Understanding the relationships between mental disorders, self-reported health outcomes and positive mental health: Findings from a national survey. Health Qual Life Outcomes. (2020) 18:55. doi: 10.1186/s12955-020-01308-0 32131837 PMC7057535

[18] Doan T Ha V Strazdins L Chateau D. Healthy minds live in healthy bodies – effect of physical health on mental health: Evidence from Australian longitudinal data. Curr Psychol. (2022) 42(22):18702–13. doi: 10.1007/s12144-022-03053-7

[19] Quanbeck C Frye M Altshuler L. Mania and the law in California: Understanding the criminalization of the mentally Ill. Am J Psychiatry. (2003) 160:1245–50. doi: 10.1176/appi.ajp.160.7.1245 12832237

[20] Flora N Barbaree H Simpson AIF Noh S McKenzie K. Pathways to forensic mental health care in Toronto: A comparison of European, African-Caribbean, and other ethnoracial groups in Toronto. Can J Psychiatry. (2012) 57:414–21. doi: 10.1177/070674371205700704 22762296

[21] Mullen PE. Forensic mental health. Br J Psychiatry. (2000) 176:307–11. doi: 10.1192/bjp.176.4.307 10827876

[22] de Mooij LD Kikkert M Theunissen J Beekman ATF de Haan L Duurkoop PWRA. Dying too soon: excess mortality in severe mental illness. Front Psychiatry. (2019) 10:855–855. doi: 10.3389/fpsyt.2019.00855 PMC6918821 31920734

[23] Eastwood MR Stiasny S Rosemary Meier HM Woogh CM. Mental illness and mortality. Compr Psychiatry. (1982) 23:377–85. doi: 10.1016/0010-440X(82)90088-8 7116833

[24] Sheidow AJ McCart M Zajac K Davis M. Prevalence and impact of substance use among emerging adults with serious mental health conditions. Psychiatr Rehabil J. (2012) 35:235–43. doi: 10.2975/35.3.2012.235.243 PMC3767039 22246122

[25] Yohanna D. Deinstitutionalization of people with mental illness: Causes and consequences. Virtual Mentor. (2013) 15:886–91. doi: 10.1001/virtualmentor.2013.15.10.mhst1-1310 24152782

[26] Ellison ML Belanger LK Niles BL Evans LC Bauer MS. Explication and definition of mental health recovery: A systematic review. Administration Policy Ment Health Ment Health Serv Res. (2018) 45:91–102. doi: 10.1007/s10488-016-0767-9 27709376

[27] Donabedian A Wheeler JRC Wyszewianski L. Quality, cost, and health: An integrative model. Med Care. (1982) 20:975–92. doi: 10.1097/00005650-198210000-00001 6813605

[28] Donabedian A. The quality of care: how can it be assessed? JAMA: J Am Med Assoc. (1988) 260:1743–8. doi: 10.1001/jama.1988.03410120089033 3045356

[29] Donabedian A. *An introduction to quality assurance in health care* - avedis donabedian - google livros. Oxford 0X2 6DP: Oxford University Press (2003).

[30] Harris MG Sparti C Scheurer R Coombs T Pirkis J Ruud T. Measurement properties of the Health of the Nation Outcome Scales (HoNOS) family of measures: Protocol for a systematic review. BMJ Open. (2018) 8. doi: 10.1136/bmjopen-2017-021177 PMC5914766 29678991

[31] Pirkis JE Burgess PM Kirk PK Dodson S Coombs TJ Williamson MK. A review of the psychometric properties of the Health of the Nation Outcome Scales (HoNOS) family of measures. Health Qual Life Outcomes. (2005) 3:76-76. doi: 10.1186/1477-7525-3-76 PMC1315350 16313678

[32] Hirdes JP Marhaba M Smith TF Clyburn L Mitchell L Lemick RA. Development of the resident assessment instrument–mental health (RAI-MH). Hosp Q. (2000) 4:2. doi: 10.12927/hcq.2000.16756 11484623

[33] Available online at: www.interRAI.org (Accessed August 01, 2024).

[34] Hirdes JP van Everdingen C Ferris J Franco-Martin M Fries BE Heikkilä J. The interRAI suite of mental health assessment instruments: an integrated system for the continuum of care. Front Psychiatry. (2020) 10:926. doi: 10.3389/fpsyt.2019.00926 32076412 PMC6978285

[35] Hirdes JP Curtin-Telegdi N Rabinowitz T Fries BE Morris JN Ikegami N. interRAI community mental health (CMH) assessment forms and user’s manual. Hirdes JP Morris JN, editors. interRAI (2010). Available at: https://catalog.interrai.org/content/interrai-community-mental-health-cmh-assessment-form-and-user%E2%80%99s-manual-standard-english (Accessed August 01, 2024).

[36] Rabinowitz T Morris JN Curtin-Telegdi N Martin L Smith TF. InterRAI emergency screener for psychiatry (ESP): Assessment forms and user’s manual (2013). Available online at: https://catalog.interrai.org/content/interrai-emergency-screener-psychiatry-esp-assessment-form-and-user%E2%80%99s-manual-standard (Accessed August 01, 2024).

[37] Stewart SL Hirdes JP Curtin-Telegdi N Perlman CM McKnight M MacLeod K. interRAI Child and Youth Heath (ChYMH) Assessment Form and User’s Manual for use with In-patient and Community-Based Assessments. Stewart S Hirdes JP, editors. interRAI (2012). Available at: https://catalog.interrai.org/ChYMH-child-and-youth-mental-health-assessment-form-and-users-manual (Accessed August 01, 2024).

[38] Hirdes JP Curtin-Telegdi N Morris JN Fries BE Rabinowitz T Perez E. interRAI mental health (MH) assessment form and user’s manual for in-patient psychiatry. Hirdes JP, editor. interRAI (2010). Available at: https://catalog.interrai.org/content/interrai-mental-health-mh-assessment-form-and-users-manual-patient-psychiatry-standard (Accessed August 01, 2024).

[39] Perlman CM Hirdes JP Barbaree H Fries BE McKillop I Morris JN. Development of mental health quality indicators (MHQIs) for inpatient psychiatry based on the interRAI mental health assessment. BMC Health Serv Res. (2013) 13:15. doi: 10.1186/1472-6963-13-15 PMC3560122 23305286

[40] Tolonen J Jaaskelainen E Leppanen V Haapea M Miettunen J Moilanen K. The impact of psychiatric rehabilitation- A study of outcomes of persons with severe mental disorders. Psychiatria Fennica. (2022) 53:204–19.

[41] Hirdes JP Curtin-Telegdi N Mathias K Perlman CM Saarela T Kolbeinson H. InterRAI mental health clinical assessment protocols (CAPs): vol. 9.1. Hirdes J, editor. Washington, DC: interRAI (2011).

[42] Ryland H Carlile J Kingdon D. A guide to outcome measurement in psychiatry. BJPsych Adv. (2021) 27:263–71. doi: 10.1192/bja.2020.58

[43] Hirdes J Ljunggren G Morris J Frijters D Finne Soveri H Gray L. Reliability of the interRAI suite of assessment instruments: A 12-country study of an integrated health information system. BMC Health Serv Res. (2008) 8:1–11. doi: 10.1186/1472-6963-8-277 19115991 PMC2631461

[44] Hirdes JP Smith TF Rabinowitz T Yamauchi K Pérez E Telegdi NC. The resident assessment instrument-mental health (RAI-MH): inter-rater reliability and convergent validity. J Behav Health Serv Res. (2002) 29:419–32. doi: 10.1007/BF02287348 12404936

[45] Almanasreh E Moles R Chen TF. Evaluation of methods used for estimating content validity. In: Research in social and administrative pharmacy. New York: Elsevier, vol. 15. (2019). doi: 10.1016/j.sapharm.2018.03.066 29606610

[46] Smith GT. On construct validity: Issues of method and measurement. psychol Assess. (2005) 17:396–408. doi: 10.1037/1040-3590.17.4.396 16393005

[47] Hanley JA McNeil BJ. The meaning and use of the area under a receiver operating characteristic (ROC) curve. Radiology. (1982) 143:29–36. doi: 10.1148/radiology.143.1.7063747 7063747

[48] Hanley JA McNeil BJ. A method of comparing the areas under receiver operating characteristic curves derived from the same cases. Radiology. (1983) 148:839–43. doi: 10.1148/radiology.148.3.6878708 6878708

[49] Sullivan GM Feinn R. Using effect size—or why the P value is not enough. J Graduate Med Educ. (2012) 4:279–82. doi: 10.4300/jgme-d-12-00156.1 PMC3444174 23997866

[50] Richardson JTE. Eta squared and partial eta squared as measures of effect size in educational research. Educ Res Rev. (2011) 6:135–47. doi: 10.1016/j.edurev.2010.12.001

[51] Cohen J. Statistical power analysis for the Behavioral Sciences. 2nd Edition. Hillsdale, N.J:Lawrence Erlbaum Associates (1988) p. 283–4.

[52] Eisen SV Dill DL Grob MC. Reliability and validity of a brief patient-report instrument for psychiatric outcome evaluation. Hosp Community Psychiatry. (1994) 45:242–7. doi: 10.1176/ps.45.3.242 8188195

[53] McKee SA Harris GT Cormier CA. Implementing residential integrated treatment for co-occurring disorders. J Dual Diagnosis. (2013) 9:249–59. doi: 10.1080/15504263.2013.807073 PMC3746518 23976887

[54] Haase RF Waechter DM Solomon GS. How significant is a significant difference? Average effect size of research in counseling psychology. J Couns Psychol. (1982) 29:58–65. doi: 10.1037/0022-0167.29.1.58

[55] Van Kranenburg GD Van Den Brink RHS Mulder WG Diekman WJ Pijnenborg GHM Mulder CL. Clinical effects and treatment outcomes of long-term compulsory in-patient treatment of treatment-resistant patients with severe mental illness and substance-use disorder. BMC Psychiatry. (2019) 19:270–270. doi: 10.1186/s12888-019-2254-9 PMC6724243 31481048

[56] Little J. Measuring and responding to mental health needs of emerging adults receiving care in Canadian Psychiatric Settings: Evidence for the importance of measuring anxiety. Waterloo, ON, Canada University of Waterloo (2022) .

[57] de Almeida Mello J Luo H Hirdes A Heikkilä J Umubyeyi B Gishoma D. An international pilot study of self-reported quality of life in outpatient and inpatient mental health settings. Front Psychiatry. (2021) 12:719994. doi: 10.3389/fpsyt.2021.719994 34421691 PMC8374624

[58] Luo H Hirdes A Heikkilä J De Cuyper K Van Audenhove C Saari M. interRAI subjective quality of life scale for mental health and addiction settings: A self-reported measure developed from a multi-national study. Front Psychiatry. (2021) 12:705415. doi: 10.3389/fpsyt.2021.705415 34305688 PMC8298814

[59] Barbaree HE Mathias K Fries BE Brown GP Stewart SL Ham E. The forensic supplement to the interRAI mental health assessment instrument: evaluation and validation of the problem behavior scale. Front Psychiatry. (2021) 12:769034. doi: 10.3389/fpsyt.2021.769034 34966306 PMC8711783

[60] Kisely S Adair CE Lin E Marriott B. Routine outcome measures in Canada. Int Rev Psychiatry. (2015) 27:286–95. doi: 10.3109/09540261.2014.994594 25738745

